# Comment on van Gemert et al. Child Abuse, Misdiagnosed by an Expertise Center—Part II—Misuse of Bayes’ Theorem. *Children* 2023, *10*, 843

**DOI:** 10.3390/children11121429

**Published:** 2024-11-26

**Authors:** Nina H. Onkenhout, Heike C. Terlingen, Michelle Nagtegaal, Elise M. van de Putte, Stephen C. Boos, Charles E. H. Berger

**Affiliations:** 1 Dutch Expertise Center for Child Abuse, 3584 EA Utrecht, The Netherlands; 2 Department of Forensic Medicine, Section of Forensic Pediatrics, Netherlands Forensic Institute, 2497 GB The Hague, The Netherlands; 3 General Pediatrics, University Medical Center Utrecht, 3584 EA Utrecht, The Netherlands; 4 Department of Pediatrics, University of Massachusetts Chan Medical School—Baystate, Baystate Medical Center, Springfield, MA 01199, USA; 5 Netherlands Forensic Institute, 2497 GB The Hague, The Netherlands; 6 Institute of Criminal Law and Criminology, Leiden University, 2311 ES Leiden, The Netherlands

## 1. Introduction

Recently, part I and part II of a series of three papers were published, namely the papers by Vlaming et al. [1] and van Gemert et al. [2]. Both papers present the case of a 2-month-old girl assessed and allegedly “misdiagnosed” by the Dutch Expertise Center for Child Abuse (DECCA; in Dutch: Landelijk Expertise Centrum Kindermishandeling—LECK). In part I of the series, the authors describe the case and discuss medico-social aspects, including the negative effects of carrying out safeguarding interventions such as foster care placement [1]. In the second paper, the authors argue that Bayes’ theorem was used incorrectly in this case and should never be used in the evaluation of cases of suspected child abuse [2].

In spite of the authors’ central allegation (as seen in their paper’s title) about the conclusion of the DECCA report, it is surprising that they never quoted the actual conclusion. DECCA receives and records patient data anonymously, and we will not comment on the details of the specific case of the 2-month-old girl discussed in the papers. With information published in the paper series, we were able to determine which DECCA report it concerned. We are therefore able to present the actual conclusion from the report, showing that the allegation was false: no “diagnosis” or probability of abuse was reported. We will also address several (mis)interpretations and methodological flaws from part II of the paper series. These inaccuracies, if left unaddressed and accepted, could have major consequences and could be harmful to children in medical, civil, and legal settings. The objective of this comment is therefore to address the false allegation, inaccuracies, and misinterpretations so as to prevent misinformation from spreading. First, we will provide some general information about DECCA and DECCA’s approach to the assessment of cases of potential child abuse. Then, we will clarify what Bayes’ theorem entails and how it should be used correctly.

## 2. General Approach of DECCA

At DECCA, specialized pediatricians, pediatric radiologists, and forensic physicians work together to offer 24/7 combined professional knowledge and experience. DECCA advises medical professionals when confronted with medical findings in potential cases of child abuse. As recommended by the Dutch reporting code for domestic violence and child abuse by the Royal Dutch Medical Association (KNMG), DECCA can be consulted in several steps of the investigation of suspected child abuse [3]. The DECCA is mostly consulted about children with injuries, such as bruises or fractures. Advisees will provide DECCA with the necessary medical information, such as photographs, radiological imaging, and laboratory results for remote review [4]. The DECCA is accessible 24/7 and gives the advisee recommendations for further diagnostics. Additionally, DECCA answers specific questions asked by the advisees—for example, about possible mechanisms of injury or about whether or not to contact the Dutch advisory and reporting center for domestic violence and child abuse (in Dutch: Veilig Thuis). These recommendations and answers are communicated to the advisee within 24 h by telephone and in writing [3].

The written reports contain evidence-based and/or expertise-based conclusions giving the evidential strength of the provided medical findings and/or injuries toward a medical condition, physiological phenomenon, or trauma (accidental or non-accidental). This evidential strength gives a certain degree of support for one hypothesis as opposed to another hypothesis. This does not equal the probability of the hypothesis being true because that probability depends on all evidence and information, including that outside the medical area of expertise. From 2019 until 2023, DECCA received over 1400 questions about medical findings or injuries in children. In 48% of cases, DECCA found that the medical findings provided evidential strength toward non-accidental causes over other causes, including accidents or illnesses. The DECCA concluded that the medical findings provided evidential strength toward other causes, including accidents and illnesses, rather than non-accidental causes in 28% of cases. In an additional 18% of cases, there was no evidential strength for either hypothesis. In 6% of cases, the evidential strength was unknown or not provided. This illustrates that the outcomes of the cases are quite varied but do usually provide evidential strength toward one of the hypotheses.

### 2.1. Point 1: The Central Problem in the Paper Series

In cases of potential child abuse, medical professionals have to weigh the risk of under-reporting against the risk of over-reporting [4]. As both of these can have serious negative effects, this can be a very difficult decision to make. In parts I and II of the paper series by Vlaming et al. and van Gemert et al., one central problem is described: to carry out safeguarding interventions while the child actually has a rare medical condition that mimics abuse. However, the authors must be aware that child abuse actually exists, and that, consequently, there is a dilemma with the following two negative possibilities:

- Risk 1: taking safeguarding measures, such as putting the child in foster care, while it may turn out that there is no actual abuse.
- Risk 2: leaving a child in a potentially dangerous situation, while investigating every possible rare condition.

The authors describe only the first and not the second risk. In reality, lives are saved by considering the second risk too. In a situation where there is some uncertainty, it is impossible to always make the right decision, in time, every time. If one follows the authors’ idea and waits until every possible rare condition (and other alternatives) could be excluded, one would indeed limit risk 1 a bit further, but only at the cost of increasing risk 2. Both risks have to be balanced, and the only way to limit one risk without raising the other is to make good expertise available where and when it is needed. This is the reason DECCA is available 24/7 and reports within 24 h, taking into account the available information at that point in time. The DECCA itself does not perform measures but provides recommendations for further diagnostics. This can help the advisee in their decision-making process to report their suspicion of child abuse to Veilig Thuis, take safeguarding measures, investigate further, or discard their suspicion of child abuse.

### 2.2. Point 2: DECCA’s Anonymous Approach

In the paper by van Gemert et al., the authors highlight the fact that “DECCA offers medical professionals anonymous advice without requesting permission from caregivers and without observing the child themselves” [2]. It is true that DECCA works with anonymous patient data. In the Netherlands, working anonymously means physicians can request advice without sharing the name of the patient. According to the Dutch reporting code, this is allowed without permission from the parents or caregivers of the child [5,6]. This approach is used to maintain a low threshold for seeking the advice of experts when physicians are faced with questions about the complexity of possible child abuse, though DECCA always encourages treating physicians to inform parents [4].

It is also true that the physicians of DECCA do not take a medical history or examine the child themselves. This does leave DECCA reliant on the history and physical examination of advisees. The DECCA does not have access to patient medical records and, sometimes, knowingly or unknowingly, has to work with incomplete information. An important reason for this approach is that the child and parents or caregivers are not burdened unnecessarily by having the same examination performed by another physician or by repeatedly having to go over the medical history. In addition, this method puts the focus on the assessment of the medical findings. Since only the information required to answer the advisee’s questions is requested by the DECCA physician, there is less risk of social and contextual bias. The advisees are ultimately responsible for providing DECCA with the necessary information. Finally, this approach allows DECCA to provide advice on a national level.

## 3. Bayesian Reasoning at DECCA

### 3.1. Point 3: The Use of Bayes’ Theorem and Likelihood Ratios at DECCA in General

For the scope of this comment, we will only provide a short overview of Bayes’ theorem and its application. We note that a theorem is, in itself, not a method or a theory but rather something that is proven from the basic laws of a science. In this case, rejecting the truth of Bayes’ theorem would require one to reject the truth of the axioms of probability theory. The experts at DECCA weigh the medical findings under one or multiple pairs of hypotheses, assessing how much evidential strength these findings provide toward one hypothesis or the other. This results in a statement about the probability of the medical findings under the hypotheses considered (often illness, a medical condition, or accidental trauma versus non-accidental trauma): a likelihood ratio. In DECCA cases, the likelihood ratio typically indicates how much more often the medical findings occur in a population of abused children than in a population of non-abused children. It is important to note that the likelihood ratio is independent of the prevalence of abuse. The likelihood ratio is usually determined from relevant scientific literature in combination with expertise from DECCA physicians.

The likelihood ratio of the medical findings is the only conclusion provided by DECCA. All other information or findings, including risk or protective factors and other context information—as combined in the prior odds—are not weighed by DECCA. The likelihood ratio multiplied by prior odds determines the posterior odds: the ratio of the probabilities of the hypotheses (e.g., non-accidental trauma versus accidental trauma) given the findings. In equation form, Bayes’ theorem states the following:

(1) prior odds×likelihood ratio=posterior odds

By using likelihood ratios as a means of reporting on just the medical findings, DECCA aims to stay within its own area of expertise and tries to remain as unbiased as possible. Reporting the conclusion as a likelihood ratio always expresses a degree of uncertainty, and the reported likelihood ratios are usually supported by scientific literature. Additionally, the use of this approach should help the experts provide conclusions that are logical, transparent, and reproducible. The method of using Bayes’ theorem in reporting has been employed by the Netherlands Forensic Institute (NFI), and in forensic science, is considered the logically correct way to report the strength of the experts’ evidence [7,8].

Van Gemert et al. do have a point when suggesting that the use of mathematical reasoning like this can cast an air of unquestionable truth over an analysis, which may exceed the actual reliability of the evidence. Unfortunately, the authors may have fallen into their own trap, as we found every calculation in their paper to be incorrect. These mistakes are important, not only in revealing the level of comprehension of the authors but also in revealing which implicit assumptions they make:

- They implicitly assume that all children in the population who have not been abused have had an accident.
- They implicitly assume that it is impossible for a physically abused child to have rib fractures.
- They implicitly assume that there is no information in the case, other than the medical findings, considered in the likelihood ratio provided by DECCA.

We will address these mistakes in the next sections.

### 3.2. Point 4: The Determination of Prior Odds

Van Gemert et al. end their methods section by estimating the incidence of physical abuse in Dutch infants and defining this as the prior odds:

(2) $$prior\;odds=\frac{P\;(physical\;abuse)}{P\;(accident)}$$

The authors estimate the rate of physical abuse in Dutch infants for P(physical abuse). They do so based on the incidence of recognized, reported, and substantiated physical abuse for children under 1 year old in the USA: 414 per 100,000 or 0.00414—obtained from National Child Abuse and Neglect Data System (NCANDS) statistics in 2020 [9]. They state that the abuse incidence in The Netherlands is more comparable to numbers from Sweden and the UK. Therefore, they adjust the physical abuse numbers from the USA with a factor of 0.211 and 0.636 for Sweden and the UK, respectively. This correction factor is based on an article by Gilbert et al. [10] from 2012. The authors conclude that the physical abuse incidence in the Netherlands is thus between 0.0009 (Sweden) and 0.0026 (UK) per year. It is unclear why they use this cumbersome method since a study on the “annual prevalence” of abuse has been performed in the Netherlands [11]. This study was published internationally and reported a physical abuse “annual prevalence” of 2.90 per 1000 (0.0029) in the Netherlands in 2017 [12]. Though “annual prevalence” is unusual terminology, both linguistically and based on the research methods, we believe this number to best approximate the incidence of physical abuse in the Netherlands. This number is close to the incidence from the UK that van Gemert et al. inferred. It should be noted that the Dutch study is based on children from all ages (under 18), that the incidence of physical abuse is much higher in the first year of life, and that these figures are for physical abuse that is recognized. As such, each of these figures likely underestimates the prior odds of child physical abuse in an infant. Additionally, we would like to point out that the definition of incidence and prevalence are not the same. Incidence gives the number of new cases over a given period of time, whereas prevalence provides the number of existing cases over a given period of time. Generally, to estimate the prior odds, prevalence is used rather than incidence. In this specific situation, for children under 1 year old in a 1-year period, we would argue they are numerically equal.

According to their own provided equation, Equation (3) in part II of the paper series, the abuse probability should be divided by the probability of an accident to convert this number to prior odds. The authors do not appear to do so (see Equations (7a) and (7b) in van Gemert et al.). They do write in their methods section: “The prior odds of abuse is the ratio of the general probability of physical abuse, here in young infants, compared to accidental causes, where these probabilities sum up to 1”. In their calculation (their Equation (3)), the authors assume P(accident) to equal 1-P(physical abuse). With their chosen probability of abuse between 0.0009 and 0.0026, P(accident) will be close to 1 if it equals 1-P(physical abuse). Theoretically, however, these probabilities do not have to add up to 1. With the abovementioned citation, the authors assume that all children who have not been abused have had an accident. The authors might have reasoned that given the injuries, a child must have either been abused or had an accident, but the injuries play no role in the prior odds, which is the very reason they are called prior odds.

In using the incidence of abuse as the prior odds, even if the calculation would have been correct, they assume that there is no other evidence and information in the case, medical or otherwise. This implicit assumption of the absence of other evidence in the case is why such calculations are not performed in DECCA reports. The DECCA only has a selection of information and thus cannot weigh all the other aspects of the case into the prior odds. Implicitly assuming the absence of any other evidence and information in the case is what happens in the well-known fallacy of the defense attorney [13].

### 3.3. Point 5: The Determination of Likelihood Ratios

In the results section, van Gemert et al. set out to define likelihood ratios for the injuries found in the case of the 2-month-old girl: bruises on varying body locations (but not on the chest) and rib fractures.

(3) $$likelihood\;ratio=\frac{P\;(injuries|physical\;abuse)}{P\;(injuries|accident)}$$

For the bruises, the authors explain that the study by Kemp et al. was used, a study of single-point prevalence of bruising in abused and non-abused children [14]. The authors state that they will assume a likelihood ratio smaller than 5 for the bruises, as 5 is the highest likelihood ratio that can be derived from the study by Kemp et al. (for the front of the trunk). They ignore that there were also locations in the study with 0 bruises in the group of abuse excluded, such as the buttocks/genitalia. On top of that, the likelihood ratio of 5 does not take into account the fact that the child in question had multiple bruises, in different locations, repeatedly, over their 2-month lifespan. It is not possible to generate another fair number without more information about the exact injuries, but the occurrence of multiple bruises at multiple times in a 2-month-old infant would make a likelihood ratio of 5 seem an underestimate. The authors wish to suggest that the number 5 is generous because Kemp et al. did not consider the possibility of other medical conditions (children with a bleeding disorder were excluded deliberately). The study by Kemp et al. certainly has some limitations, including a selected and small population in a specific setting and potential circular reasoning. The DECCA takes these limitations into account in the assignment of the evidential strength and looks at every individual case to assess what studies are applicable. Over the past few years, an increasing number of studies have been published that can be used in assigning evidential strength of bruises, for example, the studies by Kemp et al. [15] and Pierce et al. [16].

Van Gemert et al. go on to construct a likelihood ratio for the rib fractures. They accept the proposition that the mother in this case had an underlying illness, hypermobile Ehlers–Danlos Syndrome (hEDS), and assume that the child had this illness too (citing repeated bruising that continued in foster care). They determine that this resulted in reduced bone strength, which means the fractures could have been caused by childbirth or during pregnancy. We will only briefly touch upon the diagnosis of hEDS and the rib fractures. There is much more to say about the medical aspects of this case, but an in-depth analysis of the medical aspects of this case is outside the scope of this comment. Nevertheless, it should be noted that fractures during infancy as a result of hEDS are not supported by the widely accepted scientific literature [17,18]. Additionally, rib fractures as a result of childbirth are rarely seen [19,20,21,22].

Van Gemert et al. started their reasoning for their likelihood ratio by citing from the literature that compressive rib fractures are almost never accompanied by bruising. They then seem to contradict this point by assuming that the absence of bruising on the girl’s thorax excludes physical abuse as the cause. The authors come to the conclusion that the likelihood ratio for rib fractures is therefore 0. In doing this, they suggest that the probability of rib fractures in a population of abused children is 0: P(rib fracture|physical abuse) (see Equation (3)—this means it is impossible for a physically abused child to have a rib fracture. This is clearly incorrect and not how likelihood ratios are determined. Likelihood ratios rely on population data, and there is a wide range of scientific literature about rib fractures in populations of abused children [23,24].

The authors seem to criticize DECCA for coming to a final conclusion without one likelihood ratio estimate for all findings combined. They state that separate likelihood ratios must have been used, but only one likelihood ratio was mentioned in the conclusion. The DECCA usually reports separate likelihood ratios because conditional independence of the findings often cannot be assumed. Simply multiplying them would possibly overestimate the evidential strength. The DECCA does, however, try to weigh all the medical findings as a whole, using the separate likelihood ratios and their expert opinion. Usually, a very conservative approach is taken in assigning a likelihood ratio for the combination of findings. The outcome is, for example, a likelihood ratio ranging from 10 to 100.

### 3.4. Point 6: The Determination of the Posterior Odds

Van Gemert et al. rightly stressed that the likelihood ratio is not the same as the posterior odds; confusing the two is known as the fallacy of the transposed conditional, or the prosecutor’s fallacy [13]. The authors argue that DECCA computed posterior odds in this case and, thus, committed such a prosecutor’s fallacy. However, DECCA generally only reports likelihood ratios. It is up to the physician (the advisee) to combine prior odds, evidential strength from the medical findings, and any other information that weighs in to make diagnoses. The DECCA does not have all the information in the case, and there can be other findings to provide support for one of the hypotheses.

It is surprising that even as criticism is aimed at the conclusion in the DECCA report, the actual conclusion is never cited, and only paraphrased in ways that do not correspond with conclusions reported by DECCA. As mentioned before, DECCA receives and records patient data anonymously, and we will not comment on the details of the specific case discussed in the papers. The patient remains anonymous to DECCA. However, because one of the involved DECCA physicians recognized the case from the description and photographs in the papers, we were able to identify the anonymous DECCA report in question to check if a statement about the posterior odds was actually made. The conclusion of this report for a 2-month-old pre-mobile child was as follows (translated to English): “Both the bruises and rib fractures are more likely (where “more likely” is a verbal term defined as “10 to 100 times more likely” in the conclusion scale used by DECCA) under the hypothesis of a non-accidental cause than under the hypothesis of an accidental cause or illness.” Clearly, this is not a statement on the probability of the hypothesis of abuse, and therefore, not a prosecutor’s fallacy. It is also far from a decision that this would be the true hypothesis or a “diagnosis of child abuse”. Thus, the title of the paper “Child abuse, misdiagnosed by an expertise center” is incorrect and suggestive — DECCA never makes diagnoses of child abuse.

Although the authors highlight the prosecutor’s fallacy, they made this mistake themselves, several times:

1. In wrongly claiming that DECCA made this mistake. They describe in Part I of the paper series: “The expertise center was consulted again and concluded, with their protocol-based Bayesian likelihood ratios, that this case had a 10–100 times larger probability of having a non-accidental than an accidental cause.”
2. In writing: “Bayes’ statistics uses (here) the probability (P) that the infant’s bruises and rib fractures occur due to “Abuse” as the primary cause, and the probability that they occur due to an “Alternative” cause, or, using the notation P(A|B), i.e., the probability that A is observed (the infant’s bruises and rib fractures) given the assumption of B (the child has actually been physically abused or there were alternative causes)”. The first part of the sentence transposes what is correct in the second part of the sentence: it is not “the probability (P) that the infant’s bruises and rib fractures occur due to “Abuse”“, but the probability of that injury occurring if it was abuse.
3. In their Equation (12), they state: “zero abuse probability for the rib fractures”. Presumably, they think it is impossible that the rib fractures were caused by abuse; but in the equation, they put zero for the probability of observing a rib fracture if the child was abused. So, they confuse their P(abuse|rib fractures) with P(rib fractures|abuse).

### 3.5. Point 7: Should Bayes’ Theorem Be Applied in Cases of Suspected Physical Child Abuse?

Van Gemert et al. argue that “Bayesian statistics”, specifically “misused” likelihood ratios, should never be used in cases of suspected child abuse. “Bayesian statistics” actually refers to something else: frequentist statistics versus Bayesian statistics. What the authors mean is the application of Bayes’ theorem. The authors of the paper state that the values of likelihood ratios “can be very large, and physicians may therefore not feel a further need to keep searching for evidence of an accidental cause”. However, the likelihood ratios that can be derived from the literature are actually known for being quite low in the field of forensic medicine. This is partially due to the fact that medical findings often have a limited ability to help differentiate between two (generic) hypotheses. Additionally, this is due to the fact that the likelihood ratios are determined from comparison studies, meaning that likelihood ratios are also dependent on the size of the study population.

Van Gemert et al. make a valid point when observing that Bayes’ theorem is difficult to understand for non-statisticians, as was shown by previous research [25]. The DECCA is trying to identify the gaps in knowledge and improve the level of understanding of medical professionals by organizing training and education on this topic. It should be noted that the application of Bayes’ theorem is actually very similar to differential diagnostic reasoning, which is the method doctors use [26]. Consequently, the application of Bayes’ theorem might be more intuitive to doctors than van Gemert et al. suggest.

The authors also state that true cases of abuse will always be missed when using Bayes’ theorem correctly since the probability of abuse in the Netherlands is “very small”. This statement relies on the assumption that the posterior odds are determined only by the incidence/prevalence of abuse and the likelihood ratio of the medical findings. The prior odds are in fact determined by many more factors and many other pieces of “evidence” can weigh into the posterior odds.

## 4. Conclusions

Van Gemert et al. argue in their paper that DECCA misdiagnosed a case of child abuse and applied Bayes’ theorem incorrectly. While trying to explain the correct way to apply Bayes’ theorem in the case, they themselves make several mistakes, some of which we have addressed in this comment.

We regret that the persons involved have made no effort to contact DECCA if they felt like mistakes were made. The DECCA is always open to feedback and willing to engage in a constructive conversation. We hope to encourage open communication in the future.

We would like to conclude this comment by emphasizing the main goal of DECCA, which is to contribute to the protection of children by offering quick and accessible advice of high quality, based on interpretations of medical findings from a combination of pediatric and forensic expertise. The DECCA never makes diagnoses of child abuse but rather aims to only give the evidential strength—in the form of a likelihood ratio—of the medical findings as supported by the scientific literature and expertise.
